# Ultrahypofractionation of localized prostate cancer: Statement from the DEGRO working group prostate cancer

**DOI:** 10.1007/s00066-020-01723-8

**Published:** 2020-12-10

**Authors:** Frank Wolf, Felix Sedlmayer, Daniel Aebersold, Clemens Albrecht, Dirk Böhmer, Michael Flentje, Ute Ganswindt, Pirus Ghadjar, Stefan Höcht, Tobias Hölscher, Arndt-Christian Müller, Peter Niehoff, Michael Pinkawa, Nina-Sophie Schmidt-Hegemann, Constantinos Zamboglou, Daniel Zips, Thomas Wiegel

**Affiliations:** 1 Universitätsklinik für Radiotherapie und Radio-Onkologie, LKH, Universitätsklinikum, Paracelsus Medizinische Privatuniversität, Müllner Hauptstr. 48, 5020 Salzburg, Austria; 2 Universitätsklinik für Radio-Onkologie, Inselspital, Universität Bern, Bern, Switzerland; 3 Klinik für Radioonkologie und Gemeinschaftspraxis für Strahlentherapie, Klinikum Nürnberg Nord, Universitätsklinikum, Paracelsus Medizinische Privatuniversität, Nuremberg, Germany; 4 Klinik für Radioonkologie und Strahlentherapie, Charité Universitätsmedizin, Berlin, Germany; 5 Klinik und Poliklinik für Strahlentherapie, Universitätsklinikum Würzburg, Würzburg, Germany; 6 Universitätsklinik für Strahlentherapie-Radioonkologie, Innsbruck, Austria; 7 Xcare Praxis für Strahlentherapie Saarlouis, Xcare Gruppe, Saarlouis, Germany; 8 Klinik und Poliklinik für Strahlentherapie und Radioonkologie, Universitätsklinikum Carl Gustav Carus, Technische Universität Dresden, Dresden, Germany; 9 Universitätsklinik für Radioonkologie, Universitätsklinikum Tübingen, Tübingen, Germany; 10 Sana Klinikum Offenbach, Offenbach, Germany; 11 MediClin Robert Janker Klinik, Bonn, Germany; 12 Klinik für Radio-Onkologie, Universitätsklinikum der LMU, Munich, Germany; 13 Klinik für Radio-Onkologie, Universitätsklinikum Freiburg, Freiburg, Germany; 14 Abteilung Strahlentherapie, Universitätsklinikum Ulm, Ulm, Germany

**Keywords:** Extreme hypofractionation, Radiotherapy, Hypofractionation, SBRT, SABR

## Abstract

Due to its low fractionation sensitivity, also known as “alpha/beta ratio,” in relation to its surrounding organs at risk, prostate cancer is predestined for hypofractionated radiation schedules assuming an increased therapeutic ratio compared to normofractionated regimens. While moderate hypofractionation (2.2–4 Gy) has been proven to be non-inferior to normal fractionation in several large randomized trials for localized prostate cancer, level I evidence for ultrahypofractionation (>4 Gy) was lacking until recently. An accumulating body of non-randomized evidence has recently been strengthened by the publication of two randomized studies comparing ultrahypofractionation with a normofractionated schedule, i.e., the Scandinavian HYPO-RT trial by Widmark et al. and the first toxicity results of the PACE‑B trial. In this review, we aim to give a brief overview of the current evidence of ultrahypofractionation, make an overall assessment of the level of evidence, and provide recommendations and requirements that should be followed before introducing ultrahypofractionation into routine clinical use.

## Introduction

External beam radiation (EBRT) is one of the mainstays of the treatment of prostate cancer of all risk groups, to all patients who are in the decision-making process of which treatment to choose. This decision has become more complex recently, since many—equally effective—treatment alternatives are available, including active surveillance or deferred treatment for low-risk (LR) disease. The fact that LR prostate cancer bears a high risk of overtreatment is now unanimously addressed in current relevant guidelines and translates into de-escalated treatment regimens where potential side effects are very carefully weighed against the benefits of a given therapy. In contrast, high-risk (HR) prostate cancer still represents a potentially lethal disease demanding more aggressive treatment.

In this light, ultrahypofractionation qualifies as a viable option in the primary treatment of localized prostate cancer, since it can be tailored to the risk status in terms of fractional and total dose, with or without androgen deprivation therapy. In a situation where LINAC capacities are limited in many countries (or reduced as a side effect of the current COVID-19 pandemic), possibilities to reduce treatment time or fractions without compromising outcome are highly sought after. At the same time, ultrahypofractionation offers a high level of patient convenience due to low overall treatment times without an excess of toxicity. Thus, it is viewed as an attractive alternative to surgery.

In this review we recapitulate the more recent literature (randomized evidence and meta-analyses) on ultrahypofractionation, put it into context with current recommendations, and provide principles which should be followed before introducing ultrahypofractionation into clinical routine.

### Terminology

*Extreme* or *ultra*hypofractionation is commonly used synonymously with stereotactic body radiation therapy (SBRT) and stereotactic ablative body radiation (SABR), although the former terms strictly refer to the fraction size, whereas the latter also refer to the platform of beam delivery and radiation technique. We therefore chose to use the term *ultrahypofractionation* for all forms of delivery of more than 4 Gy per fraction.

The American Society for Radiation Oncology (ASTRO), American Society of Clinical Oncology (ASCO), and American Urological Association (AUA) hypofractionation guideline [1] defines moderate hypofractionation as 2.4–3.4 Gy/fraction and ultrahypofractionated radiotherapy as doses per treatment of 5.0 Gy/fraction or higher, thus leaving a “grey zone” between 3.4 and 5 Gy.

The Prostate Cancer Expert Panel of the German Society of Radiation Oncology (DEGRO) and the Working Party Radiation Oncology of the German Cancer Society (DKG-ARO) use a definition of 2.2–4 Gy/fraction for moderate and beyond 4 Gy/fraction for ultrahypofractionation [2].

Ultrahypofractionation is usually delivered using high-precision techniques (LINAC based or CyberKnife [Accuray Inc. Sunnyvale, CA, USA]) aided by daily image guidance including adequate motion management strategies allowing for small PTV margins and high dose conformation.

### Radiobiology

While for most cancer types a normofractionation schedule of 1.8–2 Gy per day/five times a week represents the sweet spot in terms of tumor control and toxicity, some tumors exhibit a higher sensitivity to fractional doses and might therefore benefit from hypofractionated schedules. This property is reflected by a low alpha/beta (α/β) value and can be quite accurately described with the so-called linear-quadratic model [3]. The α/β value is a measure of fractionation sensitivity and is related to the inherent capacity of tumor cells to repair sublethal DNA damage inflicted by ionizing radiation.

Whether hypofractionation is beneficial depends on the α/β values of the target in relation to its surrounding normal tissues. For prostate cancer cells, very low α/β values of about 1.5 Gy have been derived from multiple preclinical and clinical studies [4–9]. Late toxicity of the bladder and rectum has been estimated to have an α/β value of 5.6 Gy [10, 11] and 3 Gy [3, 12], respectively. Therefore, in theory, hypofractionated radiation schedules should have a beneficial effect on the therapeutic ratio.

More recent data have shown that in addition to fraction dose, overall treatment time seems to play a major role [13], which has been neglected in the aforementioned calculations of the α/β ratio ([14] reviewed in [15]). When a time factor is accounted for in the calculation, slightly higher α/β values will result, so that many authors nowadays endorse values of approximately 2–2.7 Gy. In a recent meta-analysis, Vogelius and Bentzen calculated α/β values based on 13 randomized trials with and without the presence of a time factor of 0.31 Gy loss per day, yielding α/β values of 1.2 Gy and 2.7 Gy, respectively. Of note, the higher α/β derived from hypofractionated dose escalation studies might in part be contributed to the fact that the dose–response relationship starts to max out at approximately EQD2 80 Gy—a dose which is superseded by most ultrahypofractionation regimens [16]. It also needs to be emphasized that when comparing EQD2’s of different fractionation regimens using the time-corrected α/β value, only regimens with the same overall treatment time should be compared. For that reason, we chose to use an α/β value of 2 Gy in the present manuscript, in order to appreciate that ultrahypofractionation regimens have a considerably reduced overall treatment time (mostly roughly 2 weeks) compared to normofractionated and moderately hypofractionated regimens.

### Cost effectiveness

In addition to its potential benefit in terms of the therapeutic ratio, ultrahypofractionation may reduce treatment cost for prostate cancer, which, due to its high prevalence, has a major impact on general health care expenses.

It has been shown that ultrahypofractionation is associated with lower overall treatment costs than normofractionated 3D conformal or IMRT [17] as well as moderate hypofractionation [18]. In a recent systemic review comprising 12 studies, Abreha et al. [19] performed a model-based cost-effectiveness analysis confirming that ultrahypofractionation is the most effective treatment in terms of overall treatment cost, including prostatectomy.

However, most available studies are based on the US Medicare system. For Europe, treatment costs can differ dramatically but there is reason to assume that the relations between different modalities remain similar [20].

### Moderate hypofractionation

For low- and intermediate-risk (IR) prostate cancer, moderate hypofractionation has been shown to be non-inferior to normofractionated treatment in several prospective randomized trials [21–24], and is now strongly recommended in the primary setting by NCCN guidelines [25] and viewed as a viable alternative in current EAU [21] and German S3 guidelines (https://www.leitlinienprogramm-onkologie.de/leitlinien/prostatakarzinom/). The latter two explicitly advise its performance only by experienced teams using high-quality EBRT (IGRT and IMRT) in carefully selected patients, with strong adherence to published phase III protocols.

For HR patients, the benefit of hypofractionated radiotherapy is less clear. Analyses from three large meta-analyses [6–8] comprising more than 20,000 patients have yielded low α/β values for all risk groups, which led Fowler et al. to conclude that “the low α/β ratio is an intrinsic property of all prostate cancer cells irrespective of their Gleason score or grading” [26]. However, clinical data to support this notion are still lacking. In the three large non-inferiority trials [22–24], HR patients were underrepresented. In the superiority design HYPRO trial [25], HR patients were included, but the primary endpoint (improved biochemical control at 5 years) did not reach significance and toxicity was slightly higher in the hypofractionated (and dose-escalated) arm.

## Literature review

### Randomized evidence

#### The Scandinavian trial (HYPO-RT-PC)

The Scandinavian non-inferiority design HYPO-RT-PC trial by Widmark et al. [26] randomized men with IR to HR prostate cancer to receive either 42.7 Gy in seven fractions, 3 days per week, or conventionally fractionated radiotherapy (78 Gy in 39 fractions, 5 days per week). After a median follow-up of 5 years, failure-free survival was identical (84%) in both arms. Acute RTOG G2 or worse genitourinary (GU) toxicity was slightly but not significantly increased in the ultrahypofractionated arm at the end of treatment (28% vs. 23%, *p* = 0.057). A significant rise was only seen at 1‑year follow-up (6% vs. 2%, *p* = 0.0037), disappearing completely at timepoints thereafter (5-year rate: 5% in both arms). There was no difference in gastrointestinal (GI) toxicity at any timepoint and no differences in toxicity after 5 years.

Of note, the Widmark trial features some peculiarities and differences compared to the abundant but retrospective trial protocols that have accumulated in the past decade. These differences need to be critically reviewed before routine clinical application:

1. It excluded LR and included intermediate- and high-risk patients: to our knowledge, the Widmark study is the only ultrahypofractionation study in which LR patients were excluded. In addition, a particular subset of HR patients (PSA <20 ng/ml and T3a) were included (11%), which is in stark contrast to the low percentage of HR patients treated within the published retrospective series.
2. It used seven instead of the commonly reported five fractions, which is unique among the ultrahypofractionation trials.
3. No androgen deprivation therapy (ADT) was given: In both arms, ADT was withheld to all patients, which might have had a negative impact on progression-free survival (PFS) as well as overall survival (OS) for (unfavorable) IR and HR patients. For these risk groups, there is no evidence that either hypofractionation or dose escalation (or both) can compensate for the lack of ADT which is known to improve both biochemical control as well as OS [27].
4. The LINAC-based radiation technique did not have to meet highest standards. Neither MR imaging for contouring nor treatment by IMRT were mandatory. In fact, 80% of patients were treated with conventional 3D planning (commented in [28]).
5. Contouring and margins: Seminal vesicles were not included in the CTV, which is questionable since EORTC guidelines recommend including the proximal 1–2 cm for IR and HR patients, respectively [29]. PTV margins were rather large (7 mm) even though image guidance was used using either BeamCath^TM^ (Beampoint AB, Kista, Sweden) (10%) or gold fiducials (90%).

#### PACE-B

Early toxicity results of the randomized PACE‑B trial have recently been published [30].

In that non-inferiority trial, men with LR or IR prostate cancer (Gleason 7b excluded) received either conventional or moderately hypofractionated radiotherapy (78 Gy in 39 fractions in 7–8 weeks or 62 Gy in 20 fractions over 4 weeks, respectively) or SBRT (36.25 Gy in five fractions over 1–2 weeks). ADT was not permitted.

A total of 41% of patients in the SBRT arm were treated with CyberKnife, 58.3% with a conventional LINAC using volumetric arc therapy (VMAT). IGRT and intra-fractional motion control were mandatory.

In terms of acute toxicity there was no significant difference between the arms, but a slight trend in favor of the SBRT arm (23% vs. 27%). This is in contrast to the Widmark trial, where a trend toward increased acute toxicity was seen in the ultrahypofractionated arm.

### Meta-analyses of non-randomized prospective data

Well over 10,000 patients have been treated within ultrahypofractionated non-randomized prospective protocols, with large variations in fraction size, total dose, and radiation technique. The most relevant studies based on quality and patient cohort size have been summarized and re-analyzed by three large pooled analyses [31–33].

#### King et al. 2013

The first pooled analysis by King et al. [31] included 1100 patients from eight institutions who had been treated within prospective phase II trials using CyberKnife with a median follow-up of 36 months. They received a median dose of 36.25 Gy in 4–5 fractions. LR (58%), IR (30%), and HR (11%) patients were included. A short course of ADT was given to 14% of patients. The five-year biochemical relapse-free survival (bRFS) rate was 93% for all patients and 95%, 84%, and 81% for LR, IR, and HR patients, respectively (*p* < 0.001). Toxicity was not reported.

#### Kishan et al. 2019

The second analysis is a cohort study Kishan et al. [32] which analyzed individual patient data from 12 phase II trials comprising 2142 men with LR and IR prostate cancer treated with either CyberKnife (7/12 studies) or a conventional LINAC (5/12 studies). 55.3% of patients had LR disease, 32.3% had favorable IR disease, and 12.4% unfavorable IR disease. HR patients were excluded. The follow-up period was quite long, with a median of 6.9 years.

Seven-year biochemical recurrence-free survival (bRFS) amounted to 95.5% for LR disease and 89.8% for IR disease. The crude incidence of acute grade 3 or higher toxic events was 0.60% for GU and 0.09% for GI side effects.

#### Jackson et al. 2019

The most recent and more extensive review was undertaken by Jackson et al. [33], comprising 6116 patients from 38 prospective studies. There was a large patient overlap with the patient collectives of the former two analyses by King and Kishan et al. A meta-analysis using random effect modeling was performed on a study-level basis. Only studies reporting the same outcome at the same timepoint were pooled, which is an inherent limitation.

At the patient level, 45% had LR, 47% had IR, and 8% HR disease. Median follow-up was 39 months, but 5‑ and 7‑year bRFS rates and toxicities were reported, not complying with the RTOG-ASTRO Phoenix consensus, which recommends the reported date of control be listed as 2 years short of the median follow-up [34].

Combined acute ≥G3 toxicity was below 1%. Late ≥G3 GU and GI toxicity was 2.0% and 1.1%, respectively, and did not change when only studies with a median FU of ≥5 years were analyzed. There was a significant publication bias, which, when corrected for, increased toxicity rates by 1 to 2%. Interestingly, there was an association of dose with late grade ≥G3 GU toxicity but not with ≥G3 GI toxicity. The authors conclude that ultrahypofractionation could be considered a standard radiotherapeutic strategy for localized prostate cancer—maybe a premature statement given how underrepresented HR patients were in that study.

### Treatment of the primary in low-volume metastatic disease setting

Two recent prospective randomized trials (HORRAD [35] and STAMPEDE [36]) have addressed the role of RT to the prostate in metastatic disease. Ultrahypofractionation is an appealing option in this scenario and has been used optionally in the STAMPEDE trial in which 48% of patients were treated with 36 Gy in 6 weekly fractions corresponding to an equivalent dose in 2Gy fractions (EQD2^α/β^ ^2^ ^Gy^) of 72 Gy. The STAMPEDE subgroup analysis of low-volume metastatic disease demonstrated a survival advantage in favor of the RT arm (hazard ratio 0.68; 95% CI 0.52–0.90). The HORRAD trial showed a similar but non-significant trend towards an improved OS by RT (hazard ratio 0.68; 95% CI 0.42–1.10). As a result, the 2019 European Association of Urology and National Comprehensive Cancer Network guidelines now include RT to the prostate as an option in the setting of low-volume metastatic disease [37].

## Interpretation

An accumulating body of retrospective evidence for the safety and efficacy of SBRT for LR and IR prostate cancer has recently been strengthened by the publication of two randomized studies comparing SBRT with a normofractionated schedule, i.e., the Scandinavian trial by Widmark et al. [26] and the first toxicity results of the PACE‑B trial [30]. Since the PACE study has not yet reached sufficient follow-up to report on outcome or late toxicity, the Scandinavian trial is thus far the only randomized trial comparing an ultrahypofractionated to a normofractionated schedule with reported long-term (i.e., 5 years) outcome and toxicity. It is therefore the only level Ib evidence (according to the Oxford Centre for Evidence-Based Medicine, https://www.cebm.net/index.aspx?o=5653) available and has raised the grade of recommendation from C to B. This could justify the clinical use of ultrahypofractionation outside of clinical trials for low- and intermediate-risk prostate cancer.

HR patients should continue to be treated within clinical trials due to several reasons: First, the body of evidence for LR and IR prostate cancer is overwhelmingly larger than for HR prostate cancer patients constituting well below 8% of studied patients. In addition, it remains disputable whether Gleason 8–10 prostate cancer cells feature an equally low α/β value, although this has been postulated [38]. Third, ultrahypofractionation of the prostate hampers simultaneous coverage of pelvic lymph nodes. Pelvic RT in HR patients is endorsed by many institutions, although its value is still controversially discussed. However, an ultrahypofractionated boost with reduced dose after whole pelvic RT might be an attractive concept [39, 40].

ADT has been used inconsistently in the available ultrahypofractionation trials. However, at the present time there is no evidence that high dose can compensate for the lack of ADT in higher-risk prostate cancer. Therefore, ADT should be administered according to the current guidelines, i.e., short-term ADT (4–6 months) for unfavorable IR and long-term ADT (18–36 months) for HR prostate cancer. For favorable IR the omission of ADT seems appropriate in a dose-escalated setting [27].

For ultrahypofractionation, the ideal fractionation regimen has not yet been established. A variety of schedules have been published and can be considered safe (see Table 1 for select examples and corresponding EQD2s for different α/β values). With the exception of the Scandinavian trial by Widmark et al., most larger series used doses of 36.25 to 40 Gy in five fractions administered every other day. In the meta-analysis by Jackson et al., the median fraction size was 7.4 Gy. For unfavorable IR patients, slightly higher total doses may be considered. The seven-fraction schedule by Widmark et al. has been tested in a randomized prospective fashion and can be considered a viable option for IR patients.

**Table 1:** Treatment schedules and corresponding EQD2 for different α/β values

	Dose/fx	No. fx	Total dose	EQD2 α/β 1.5	EQD2 α/β 2	EQD2 α/β 3	EQD2 α/β 10
HYPO-RT-PC [26]	6.1	7	42.7	92.72	86.47	77.71	57.29
PACE [30]	7.25	5	36.25	90.63	83.83	74.31	52.11
Median fx Jackson et al. [33]	7.4	5	37	94.09	86.95	76.96	53.65
STAMPEDE primary metastatic [36]	6	6	36	77.14	72.00	64.80	48.00

*fx* fraction, *EQD2* equivalent dose in 2Gy fractions

For treatment of the primary in the metastatic setting, ultrahypofractionation is an attractive alternative to moderate hypofractionation and has been tested in the STAMPEDE trial. Prolongation of the overall treatment time to 6 weeks is rather unusual for an ultrahypofractionated regimen and may be the reason why in this trial, the alternatively used moderately hypofractionated radiation schedule of 55 Gy in 20 fractions (daily) showed a slightly better outcome (not significant, HR 0.86 vs. 1.01).

Compared to radiation schedules in the non-metastatic setting, the resulting BEDs of these doses are rather conservative, which owes to the palliative setting where the maxim *primum non nocere* is to be followed strictly. In general, further studies are needed to establish an appropriate fractionation schedule in the metastasized setting, but if ultrahypofractionation is used, the DEGRO Prostate Cancer Expert Panel favors every-other-day schedules over once-weekly regimens and recommends aiming for a total dose of at least EQD2^α/β2^ 72 Gy.

## Conclusion

Retrospective as well as randomized prospective data with a follow-up of 5 years or more are now available and have shown comparable results to recent moderate hypofractionation trials in terms of both biochemical control and toxicity. Although level Ib evidence for ultrahypofractionation is now available, the DEGRO prostate cancer expert panel does not yet recommend ultrahypofractionation for HR patients on a routine basis. However, for centers that are experienced in SBRT and wish to offer ultrahypofractionation to selected LR and IR patients, this seems justified outside of a clinical trial (grade B recommendation).

In interpretation of the published data, the following principles should be followed when administering ultrahypofractionation in prostate cancer in clinical routine.

1. Ultrahypofractionation is a treatment alternative, amongst moderate hypofractionation and normofractionation, which can be offered outside clinical trials to LR patients who are not suitable for active surveillance and for IR patients including both favorable and unfavorable IR.
2. ADT should be administered according to current guidelines for normo- and moderately hypofractionated regimens, i.e., short-term ADT for unfavorable IR (and long-term ADT for HR patients). For favorable IR, dose-escalated RT alone appears to be an appropriate treatment.
3. Ultrahypofractionation should be administered in a setting of high technical standards. We consider MR-based planning, IMRT/VMAT, and daily IGRT with basic intrafractional control (i.e., imaging after 3 min of treatment time) as minimum requirements in order to safely achieve PTV margins of approximately 3 mm. In a LINAC-based setting, short treatment times need to be pursued using single-arc VMAT and/or flattening filter-free (FFF) techniques.
4. Dose schedules should strictly adhere to published concepts of larger studies. A maximum fraction size of 8 Gy and a maximum EQD2^α/β2^ of 100 Gy should not be exceeded. In the curative, non-metastatic setting, a minimum EQD2^α/β2^ of 83.3 Gy (e.g., 5 × 7.25 Gy) is required. See Table 1 for different fractionation schedules and corresponding EQD2s at different α/β values.
5. For treatment of the primary in the metastatic setting, ultrahypofractionation may be used as an alternative to moderate hypofractionation. However, slightly de-escalated schedules with an EQD2^α/β2^ of approximately 72–76 Gy should be used.
6. Patients should be followed up by the treating facility for at least 5 years. Inclusion in a registry study is recommended.
